# Glibenclamide for the Treatment of Ischemic and Hemorrhagic Stroke

**DOI:** 10.3390/ijms16034973

**Published:** 2015-03-04

**Authors:** Nicholas Caffes, David B. Kurland, Volodymyr Gerzanich, J. Marc Simard

**Affiliations:** 1 Department of Neurosurgery, University of Maryland School of Medicine, Baltimore, MD 21201, USA; E-Mails: nicholas.caffes@som.umaryland.edu (N.C.); kurland.davidb@gmail.com (D.B.K.); vgerzanich@smail.umaryland.edu (V.G.); 2 Department of Pathology, University of Maryland School of Medicine, Baltimore, MD 21201, USA; 3 Department of Physiology, University of Maryland School of Medicine, Baltimore, MD 21201, USA

**Keywords:** ischemic stroke, subarachnoid hemorrhage, Sur1-Trpm4 channels, K_ATP_ channels, glibenclamide, cerebral edema, necrotic cell death

## Abstract

Ischemic and hemorrhagic strokes are associated with severe functional disability and high mortality. Except for recombinant tissue plasminogen activator, therapies targeting the underlying pathophysiology of central nervous system (CNS) ischemia and hemorrhage are strikingly lacking. Sur1-regulated channels play essential roles in necrotic cell death and cerebral edema following ischemic insults, and in neuroinflammation after hemorrhagic injuries. Inhibiting endothelial, neuronal, astrocytic and oligodendroglial sulfonylurea receptor 1–transient receptor potential melastatin 4 (Sur1–Trpm4) channels and, in some cases, microglial K_ATP_ (Sur1–Kir6.2) channels, with glibenclamide is protective in a variety of contexts. Robust preclinical studies have shown that glibenclamide and other sulfonylurea agents reduce infarct volumes, edema and hemorrhagic conversion, and improve outcomes in rodent models of ischemic stroke. Retrospective studies suggest that diabetic patients on sulfonylurea drugs at stroke presentation fare better if they continue on drug. Additional laboratory investigations have implicated Sur1 in the pathophysiology of hemorrhagic CNS insults. In clinically relevant models of subarachnoid hemorrhage, glibenclamide reduces adverse neuroinflammatory and behavioral outcomes. Here, we provide an overview of the preclinical studies of glibenclamide therapy for CNS ischemia and hemorrhage, discuss the available data from clinical investigations, and conclude with promising preclinical results that suggest glibenclamide may be an effective therapeutic option for ischemic and hemorrhagic stroke.

## 1. Introduction

Each year, 5.5 million people die from an ischemic stroke, with 10%–12% suffering from “malignant infarctions”, rapidly progressing cerebral edema that compromises arterial inflow, culminating in further ischemic damage [1]. The prognosis for these patients is poor, with mortality rates as high as 60%–80% [1,2]. The only medication currently approved specifically for use in acute ischemic stroke is recombinant tissue plasminogen activator (rtPA); however, for many reasons, it is used in <20% of stroke victims, even in societies with the most advanced healthcare [3,4,5,6]. Additional standards of care focus on treating brain swelling after it becomes symptomatic. Life-saving decompressive craniectomy, as well as largely unproven drug interventions such as mannitol and hypertonic saline, are the mainstays of treatment today, with novel therapies aimed at preventing cerebral edema severely lacking.

Hemorrhagic stroke, while less prevalent than ischemic stroke, is a devastating injury that accounts for 15% of strokes annually in the United States [7]. Current therapies are largely supportive, consisting of blood pressure control, reversal of bleeding diatheses, and surgical or endovascular management. Central nervous system (CNS) injury is often exacerbated, in part due to the robust neuroinflammtory responses to extravasated blood and blood products. Novel strategies to target and reduce neuroinflammation after intracerebral hemorrhage (ICH) hold the promise of reducing the burden of this disease. Activation of sulfonylurea receptor 1 (Sur1) regulated channels has been identified as a key molecular mechanism of cerebral edema following ischemic insults and, intriguingly, Sur1 inhibition has demonstrated novel protective anti-inflammatory effects in pre-clinical models of subarachnoid hemorrhage [8]. Targeted inhibition of Sur1-regulated channels by the sulfonylurea glibenclamide (also known as glyburide, US adopted name) may offer an effective new treatment option for both ischemic and hemorrhagic forms of stroke.

Glibenclamide is a member of the sulfonylurea class of drugs whose therapeutic benefits as oral hypoglycemic agents date back to the 1960s [9]. Sulfonylurea drugs work via inhibition of Sur1. Patients with diabetes mellitus type II (DM II) benefit from glibenclamide treatment via inhibition of K_ATP_ (Sur1–Kir6.2) channels in pancreatic β islet cells, leading to increased insulin release [10,11,12,13]. With its long history of safety and efficacy in treating DM II, glibenclamide has provided the foundation upon which newer diabetic mono- and combined therapies have been developed [14].

During the last decade, glibenclamide has received renewed attention due to its pleiotropic protective effects in acute CNS injury. In the CNS, glibenclamide primarily inhibits the recently characterized sulfonylurea receptor 1–transient receptor potential melastatin 4 (Sur1–Trpm4) channel [15] and, in some cases, microglial Sur1–Kir6.2 (K_ATP_) channels [16,17]. Several preclinical studies have found glibenclamide to be an effective treatment in rodent models of ischemic stroke [16,17,18,19,20,21,22,23,24], and retrospective studies suggest that being on a sulfonylurea drug and staying on it following ischemic CNS insults significantly improves outcomes [25,26]. The successes of these preclinical experiments [27] have set the stage for clinical trials examining glibenclamide’s protective effects following ischemic strokes [28,29,30].

Additional laboratory investigations have implicated Sur1 in the pathophysiology of hemorrhagic CNS insults, particularly in the development of vasogenic edema and neuroinflammation. In clinically relevant models of subarachnoid hemorrhage, glibenclamide ameliorated several adverse short-term outcomes and, more importantly, improved long-term cognitive function [31,32].

Recent publications have reviewed the roles of Sur1 [33], Trpm4 [34] and K_ATP_ [35,36,37] channels in CNS injury. However, our purpose in this review is to highlight the potential uses for glibenclamide in treating ischemic CNS insults and to present preclinical evidence supporting glibenclamide’s therapeutic potential in hemorrhagic CNS lesions. We provide an overview of the preclinical studies of glibenclamide therapy for CNS ischemia, discuss the available data from clinical investigations, and conclude with promising preclinical results that suggest glibenclamide may be an effective therapeutic option for ischemic and hemorrhagic stroke.

## 2. Mechanisms of Sulfonylurea Receptor 1 (Sur1) Pathology

Sur1 is encoded by the *Abcc8* gene and acts as the regulatory subunit for two distinct ion channels: (i) the ATP-sensitive K^+^ channel, Kir6.2, which, together with Sur1, forms K_ATP_ channels [38,39,40]; and (ii) the ATP- and calcium-sensitive non-selective cation channel, transient receptor potential melastatin 4 (Trpm4), which, together with Sur1, forms Sur1–Trpm4 channels [15]. K_ATP_ and Sur1–Trpm4 channels, while regulated by Sur1, have opposite functional effects. Opening of K_ATP_ channels hyperpolarizes the cell [36] whereas opening of Sur1–Trpm4 channels depolarizes the cell. Cell depolarization or hyperpolarization has important physiological consequences. Sur1–Trpm4-mediated depolarization is important for reducing pathological calcium influx via voltage-independent channels, but if unchecked, ion flow through these channels causes cytotoxic edema and necrotic cell death [33,34]. K_ATP_ mediated hyperpolarization is important for reducing calcium influx via voltage-dependent channels, but when excessive, exhausts ATP consuming compensatory measures in neurons [41] and blunts cellular responses to external stimuli in microglia [16].

Sur1–Trpm4 channels in neurons, astrocytes, oligodendrocytes, and microvascular endothelial cells are upregulated after focal ischemia [18,42] and hemorrhage [8], presumably to protect against an excessive rise in intracellular calcium [15,33] and subsequent triggering of calcium-dependent cell death cascades [43,44]. However, extreme depletion of ATP, as occurs in ischemia and hemorrhage, can result in persistent channel activation leading to the pathological influx of Na^+^, Cl^−^, and water, providing a major molecular mechanism of cytotoxic edema and necrotic (oncotic) cell death in the CNS [18,34,45].

While pathological involvement of Sur1–Trpm4 channels has been demonstrated in ischemic and hemorrhagic CNS injury, recent evidence also supports a potential role of brain K_ATP_ channels in promoting neuroglial injury. In ischemia, ATP depletion results in excessive neuronal K_ATP_ mediated potassium efflux, which may increase the electrochemical driving force for and subsequent influx of calcium, a key regulator of cell death cascades [41]. Microglial K_ATP_ mediated potassium efflux may also result in potent disturbances in membrane potential and interfere with favorable microglial responses to the surrounding neurochemical milieu. Indeed, recent evidence links ischemia induced K_ATP_ channel activation to the development of neurotoxic microglial phenotypes [16,17].

Of note, these Sur1-regulated channels are transcriptionally upregulated progressively during several hours after the onset of ischemia or hemorrhage [46]. Critically, because hours pass between the CNS insult and Sur1 upregulation, a very favorable therapeutic time window exists to target and prevent Sur1-mediated CNS damage.

## 3. Glibenclamide Uptake in Central Nervous System (CNS) Hemorrhage and Ischemia

The Sur1-Trpm4 channel is blocked by first and second-generation sulfonylureas. Normally, glibenclamide does not accumulate in the brain [47]. However, penetration into the brain is enhanced after ischemic and hemorrhagic insults. Brain ischemia results in focal lactic acidosis and a relatively low pH environment [48]. Glibenclamide is a weak acid and, as such, its lipid solubility and ability to penetrate the blood-brain barrier (BBB) is enhanced at low pH. In the context of CNS hemorrhage, the dysfunctional BBB enhances the passive uptake of glibenclamide into tissues localized to the injury focus [33]. With local BBB breakdown, plasma extravasation leads to vasogenic edema, which carries glibenclamide, a highly protein bound drug, into the extravascular space. As a result, relatively low doses of drug can be used to obtain a favorable therapeutic effect in both ischemic and hemorrhagic stroke [33].

## 4. Ischemic Stroke

### 4.1. Targeting Sur1 in CNS Ischemia—Animal Models

Focal CNS ischemia is associated with progressive microvascular dysfunction. This dysfunction manifests initially as ionic edema, which may be followed by vasogenic edema and “hemorrhagic transformation”, depending on the severity of the initial insult [49]. Further secondary injury can occur via compression of adjacent tissues, ultimately leading to death. While complex, a key molecular event involved in this microvascular dysfunction is upregulation of Sur1-regulated channels and subsequent damage mediated by these channels. Preclinical studies from several independent laboratories have demonstrated that Sur1 inhibition by glibenclamide reduces adverse secondary manifestations and increases favorable outcomes in rat models of focal cerebral ischemia.

### 4.2. Glibenclamide Inhibition of Sur1 in Non-Lethal Stroke

In several different models of moderate-severity ischemic stroke, sulfonylurea therapy favorably modulates CNS injury, neurogenesis, and long-term neurological function. In non-lethal stroke models, glibenclamide or gliclazide reduce lesion volume and cortical damage and improves functional neurological scores [17,19,23,24]. Glibenclamide treatment after focal ischemia also increases BrdU and NeuN labeling in and around areas of infarction, suggesting enhanced cortical neurogenesis [17]. The long-term effects of glibenclamide treatment after non-lethal ischemia also have been examined. Ortega *et al.* [16,17] found significant improvements in sensorimotor and cognitive functions lasting up to 1 month following glibenclamide therapy for ischemic CNS insults. In these studies by Ortega and colleagues [16,17] as well as in a recent review [50], glibenclamide was postulated to exert its long term protective effect following ischemia via inhibition of microglial K_ATP_ channels, thereby inducing a neuroprotective phenotype characterized by an increased microglial phagocytic capacity.

### 4.3. Glibenclamide Inhibition of Sur1 in Lethal Stroke

Glibenclamide is also a potent therapeutic agent in cerebral ischemia complicated by “malignant” cerebral edema. In models of malignant infarction, glibenclamide significantly reduces edema formation, hemispheric swelling, and mortality [18,20,22]. Compared to decompressive craniectomy (DC), which is often performed on patients with severe stroke complicated by malignant edema [51], glibenclamide reduced brain swelling and improved neurological outcome [20]. Interestingly, both DC and glibenclamide eliminated mortality, but neurological function during subsequent weeks was only improved with glibenclamide, suggesting that preventing swelling is preferable to decompressing the already swollen brain [20].

### 4.4. Glibenclamide vs. Recombinant Tissue Plasminogen Activator (rtPA)

Currently, the only drug specifically approved for the treatment for acute ischemic stroke is recombinant tissue plasminogen activator (rtPA). However, some risks, particularly hemorrhagic conversion, accompany its use [52]. In a rat model of ischemia, glibenclamide, when combined with rtPA, reduced symptomatic hemorrhagic transformation and edema. While solitary therapy with glibencalmide or rtPA improved neurological outcomes, the best scores were observed in rats treated with both rtPA and glibenclamide [21,22].

### 4.5. Retrospective Clinical Studies

Patients with diabetes mellitus type 2 (DM-2) managed with a second generation sulfonylurea drug were compared to those with diabetes managed with diet and exercise alone following an acute ischemic stroke to determine if sulfonylurea treatment attenuated the neurological damage inflicted by CNS ischemia. Two reports [53,54] compared diabetic patients who were using a sulfonylurea *prior* to stroke onset to those who were not, and they reported no adverse effects on stroke severity, mortality, or long-term functional outcomes with prior sulfonylurea use. Moreover, they observed that subsets of patients who continued sulfonylurea therapy exhibited mild neurological improvement. Follow-up studies [25,26] retrospectively compared diabetics who were not on a sulfonylurea to those who were during the days following acute ischemic strokes, finding a strong association between sulfonylurea treatment and improved survival, greater functional independence, lower NIH stroke scale scores, and less hemorrhagic transformation. Together, these retrospective studies of diabetic patients presenting with stroke suggest that if a patient is on a sulfonylurea drug at stroke onset, this drug should be continued unless contraindicated.

### 4.6. Prospective Clinical Studies

The preclinical evidence supporting the potential of glibenclamide to ameliorate detrimental secondary manifestations and improve neurological outcomes in rat models of ischemic stroke, coupled with the retrospective human studies reviewed above, have led to the initiation of prospective clinical trials. These prospective trials are evaluating the therapeutic effects of RP-1127, an IV formulation of glibenclamide.

Recently, a Phase IIa study was completed that evaluated the potential benefit of RP-1127 in 10 patients with a severe anterior circulation ischemic stroke at high risk for malignant cerebral edema [28,29,30]. Compared to an untreated comparison cohort, glibenclamide reduced the incidence of malignant edema, and only 2/10 patients required current standard therapies such as osmotherapy, intubation, or decompressive craniectomy. Clinically significant parenchymal hematomas were reduced from an anticipated rate of ~30% to 0%. Compared to a pooled analysis of decompressive craniectomy trials [55], the proportion of patients with 30-day modified Rankin Scale scores (mRS) ≤4 was increased with RP-1127 therapy. Currently, a larger clinical trial is underway to demonstrate the safety and efficacy of IV RP-1127 following severe anterior circulation ischemic strokes.

## 5. Sur1 in Hemorrhagic Stroke

### 5.1. Subarachnoid Hemorrhage

Subarachnoid hemorrhage (SAH) accounts for 10% of the overall stroke burden in society and has a 30-day mortality rate approaching 50%. Those who survive experience secondary injury and suffer long-term cognitive disability due to a variety of injury cascades including oxidative stress, neuroinflammation, and vasospasm [56,57,58]. While vasospasm has long been considered the major cause of secondary injury, recent studies indicate that reversing vasoconstriction is not necessarily associated with improved clinical outcome [59]. Inflammation following SAH can also induce secondary brain injury, and may itself be a cause of vasospasm. Inflammation induces apoptosis and alters the blood-brain barrier leading to increased permeability and subsequent vasogenic edema [60,61]. Interestingly, the 5'-flanking region of the *Abcc8 promoter*, the gene that encodes Sur1, contains two consensus binding sites for nuclear factor κB (NF-κB), suggesting that Sur1 may participate in the inflammatory response following SAH [31].

### 5.2. Targeting Sur1 in Subarachnoid Hemorrhage

In the proinflammatory context of SAH induced by unilateral puncture of the internal carotid artery, *in situ* hybridization showed strong expression of *Abcc8* mRNA and immunohistochemistry demonstrated abundant expression of Sur1 in neurons and microvessels adjacent to the SAH, where TNFα and NF-κB signaling were maximal. *In vitro* experiments confirmed the association between inflammation and Sur1, showing that Sur1 upregulation was induced by TNFα-mediated activation of NF-κB. Additional studies implicated Sur1-Trpm4 in the pathophysiology of SAH. In the brains of humans and rats after SAH, co-associated Sur1 and Trpm4 subunits, but not Sur1 and Kir6.2 subunits, were readily detectable in cortical areas adjacent to the area of hemorrhage [8].

The effects of Sur1 inhibition with glibenclamide following SAH also have been examined. Glibenclamide reduced vasogenic edema, inflammation, and caspase-3 activation after SAH [31]. SAH causes a large increase in BBB permeability and disrupts the normal expression and localization of tight junction proteins. Glibenclamide significantly attenuated the SAH-induced alteration in BBB permeability, as demonstrated by reduced tight junction abnormalities and reduced edema formation. SAH also results in the accumulation of blood-borne substances in brain parenchyma, resulting in the activation of microglia and astrocytes and amplification of the inflammatory response. In rats treated with glibenclamide after SAH, local inflammation and reactive astrocytosis were significantly reduced compared to vehicle treated rats [31]. This reduction in inflammation had a significant effect on cell death. Inflammation often results in activation of signaling pathways that induce apoptosis, particularly via TNFα induced activation of caspase-3. In animals administered glibenclamide, caspase-3 activation and resulting apoptosis were significantly reduced in endothelial and parenchymal cells.

While our understanding of the pathological effects of inflammation and vasospasm in the context of SAH is improving, there has been a paucity of attention focused on therapies to improve cognitive impairment following SAH. In rats, the neuronal circuitry involving entorhinal cortices plays a critical role in spatial learning [62], and hemorrhage here mimics high grade SAH that often leaves patients with long-term cognitive impairments. Following entorhinal SAH, animal models revealed extensive neuroinflammation, demyelination, and apoptosis [63], which lead to deficits in spatial learning tasks [8]. Glibenclamide treatment, however, reduced apoptosis of hippocampal neurons, preserve white matter in the perforant pathway and, most importantly, significantly ameliorated long-term impairments in spatial learning [8].

The evidence reviewed above supports the role of Sur1-regulated channels in the pathophysiology of SAH and the potential for glibenclamide to ameliorate several short-term adverse effects, including BBB disruption, inflammation, and caspase-3 activation. Most importantly, by targeting the underlying inflammatory process responsible for secondary brain injury following SAH, glibenclamide appears to improve long-term cognitive outcomes.

## 6. Conclusions

The protective effects of glibenclamide have been established in clinically relevant rodent models of ischemic and hemorrhagic CNS insults. Emerging evidence points to a critical role for Sur1-Trpm4 channels in cytotoxic (cellular) edema, necrotic cell death, microvascular dysfunction, ionic and vasogenic edema formation, secondary hemorrhage and neuroinflammation. Additional evidence points to a critical role for K_ATP_ (Sur1-Kir6.2) channels in microglial reactivity. As part of these seminal advances, glibenclamide has been shown to be highly beneficial for reducing CNS damage and improving outcomes in non-lethal and lethal rat models of ischemic and hemorrhagic stroke. Retrospective studies, as well as a prospective Phase IIa pilot study, suggest a highly promising translational potential for therapeutic intervention with glibenclamide in ischemic stroke. Together, these studies have catalyzed our understanding of Sur1 in ischemic and hemorrhagic CNS insults. Glibenclamide shows great promise for the future treatment of two devastating CNS insults, providing a much needed option for diseases where limited therapies currently exist.
